# Automated tumor proportion score analysis for PD-L1 (22C3) expression in lung squamous cell carcinoma

**DOI:** 10.1038/s41598-021-95372-1

**Published:** 2021-08-05

**Authors:** Jingxin Liu, Qiang Zheng, Xiao Mu, Yanfei Zuo, Bo Xu, Yan Jin, Yue Wang, Hua Tian, Yongguo Yang, Qianqian Xue, Ziling Huang, Lijun Chen, Bin Gu, Xianxu Hou, Linlin Shen, Yan Guo, Yuan Li

**Affiliations:** 1 Department of Pathology, Fudan University Shanghai Cancer Center, Shanghai, China; 2 Department of Oncology, Shanghai Medical College, Fudan University, Shanghai, China; 3 Histo Pathology Diagnostic Center, Shanghai, China; 4 Department of Pathology, Yangzhou Jiangdu People’s Hospital, Yangzhou, China; 5 Computer Vision Institute, School of Computer Science and Software Engineering, Shenzhen University, Shenzhen, China; 6 AI Research Center for Medical Image Analysis and Diagnosis, Shenzhen University, Shenzhen, China

**Keywords:** Image processing, Machine learning, Diagnostic markers, Non-small-cell lung cancer, Computational models, Computational biology and bioinformatics

## Abstract

Programmed cell death ligend-1 (PD-L1) expression by immunohistochemistry (IHC) assays is a predictive marker of anti-PD-1/PD-L1 therapy response. With the popularity of anti-PD-1/PD-L1 inhibitor drugs, quantitative assessment of PD-L1 expression becomes a new labor for pathologists. Manually counting the PD-L1 positive stained tumor cells is an obviously subjective and time-consuming process. In this paper, we developed a new computer aided Automated Tumor Proportion Scoring System (ATPSS) to determine the comparability of image analysis with pathologist scores. A three-stage process was performed using both image processing and deep learning techniques to mimic the actual diagnostic flow of the pathologists. We conducted a multi-reader multi-case study to evaluate the agreement between pathologists and ATPSS. Fifty-one surgically resected lung squamous cell carcinoma were prepared and stained using the Dako PD-L1 (22C3) assay, and six pathologists with different experience levels were involved in this study. The TPS predicted by the proposed model had high and statistically significant correlation with sub-specialty pathologists’ scores with Mean Absolute Error (MAE) of 8.65 (95% confidence interval (CI): 6.42–10.90) and Pearson Correlation Coefficient (PCC) of 0.9436 ($p < 0.001$), and the performance on PD-L1 positive cases achieved by our method surpassed that of non-subspecialty and trainee pathologists. Those experimental results indicate that the proposed automated system can be a powerful tool to improve the PD-L1 TPS assessment of pathologists.

## Introduction

The programmed cell death-1 (PD-1) and its ligands of PD-L1 and PD-L2, known as a family of immune checkpoint proteins, act as T-cell co-inhibitory factors, which is able to suppress the immune response. The interaction of PD-1 and PD-L1 ensures that the immune system is activated at the appropriate time^1^. PD-L1 expressed on tumor cells bind to PD-1 receptors on the activated T-cells, which helps tumor cells evade anti-cancer immunity^2,3^. Monoclonal antibodies that blockade the interaction between PD-1 and PD-L1 can restore the ability of immune response to kill cancer cells^4,5^. According to this mechanism of action (MOA), a number of inhibitors have been developed. Existing immunotherapeutic drugs involving anti-PD-1 inhibitor and anti-PD-L1 inhibitor have provided promising results in clinical trials^6,7^.

Different PD-L1 IHC assays have been co-developed as companion or complementary diagnostics to different anti-PD-1/PD-L1 inhibitor drugs^8^. The 28-8 antibody have been approved by both U.S. Food and Drug Administration (FDA) and National Medical Products Administration (NMPA) China as a complementary diagnostic to nivolumab for non-small cell lung cancer (NSCLC). The pembrolizumab uses the 22C3 antibody as a companion diagnostic assay^9^. Most recently, FDA has expanded the use of the 22C3 assay alongside cemiplimab-rwlc for advanced NSCLC. Higher tumoral expression of PD-L1 evaluated by immunohistochemical (IHC) assays has been approved to be associated with remarkably longer overall survival and with fewer adverse events on NSCLC^10–12^.

The Dako PD-L1 IHC 22C3 and 28-8 pharmaDx assays employ tumor proportion score (TPS) to measure PD-L1 expression. The TPS is determined as the percentage of PD-L1 positive stained tumor cells (TCs) with at least partial membrane staining relative to the total number of TCs, excluding tumor-associated interstitial cells (ICs), necrotic, normal or non-neoplastic cells from the evaluation:

$$\begin{aligned} TPS(\%) = \frac{ \# \text{ of } \text{ PD-L1 } \text{ positive } \text{ TCs }}{ \text{ Total } \# \text{ of } \text{ viable } \text{ TCs }} \times 100. \end{aligned}$$

Therefore, the pathological assessment of tumoral PD-L1 expression is based on two semi-quantitative information of PD-L1 positive tumor cell number and total viable tumor cell number.

In clinical practice, pathologists determine the TPS by microscopic examinations. Specifically, when the slide has a single PD-L1 positive tumor area, the TPS is determined as the product of the % of positive staining area and the % of positive TCs in the area. Whereas for the slide with heterogeneous tumor areas, TPS is calculated by averaging the stained tumor cell percentages of several divided tumor areas^13^. Manually assessment is obviously time-consuming and subjective. It is hardly possible for pathologists to conduct visually quantitative analysis based on the whole slide images (WSI), which may contain millions of cells. Furthermore, imprecise and subjective definition of stain intensity makes the assessment even more difficult to ensure the inter- and intra-observer reproducibility.

With the advent of whole slide imaging scanners and dramatic improvements in computer vision algorithms, an increasing number of clinicopathologic Computer Aided Diagnosis (CAD) systems have been proposed^14^. Previous works have shown promising performance in many straightforward tasks, such as metastasis detection^15^, tumor region detection^16,17^, and cell detection^18^, etc. Researchers also introduced high level assessment system based on comprehensive information. Liu et al. proposed an end-to-end deep learning framework for automatic histochemical-score assessment for breast cancer tissue microarray^19^. A deep reinforcement learning based model was introduced for HER2 scoring, which was able to select diagnostically relevant regions^20^. Christiansen et al. showed their pioneering research of predicting fluorescent labels from transmitted-light images of unlabeled samples^21^ using supervised machine learning techniques. Inter- and intra-observer concordances are essential and widely used for CAD system evaluation. Luo et al. compared their proposed gastrointestinal artificial intelligence diagnostic system with three endoscopists of varying degrees of expertise^22^. Eleven pathologists of four experience levels were involved to assess the deep learning assistant system^23^.

Given the recent advances in the field of deep learning and computer vision, researchers have proposed several deep learning based frameworks for automatic PD-L1 scoring^24,25^. However, prior literature has focused on the methodology. In this study, we introduced computer aided TPS system named Automated Tumor Proportion Scoring System (ATPSS) for PD-L1 tumor proportion score assessment on lung squamous cell carcinoma slides. The system was designed to mimic the scoring process of the pathologists by only using PD-L1 stained IHC digital slides as input. We first constructed a tumor image and a positive tumor image, which were produced by deep learning based model of *tumor area segmentation* and image processing based module of *positive membrane detection*. The numbers of total TCs and PD-L1 positive TCs were counted on those two images by *nuclei detection* model for TPS calculation. To get a better understanding of AI aided PD-L1 TPS system, we conducted a multi-reader multi-case study. The main contribution of this paper is threefold and summarized as follows:

- To the best of our knowledge, this is the first comprehensive study involving different experience level pathologists and different PD-L1 expression level cases for the automatic TPS system evaluation.
- The proposed system was designed with recently published deep learning techniques and trained with the simplest labels of tumor region and tumor nuclei center.
- The results demonstrated that the ATPSS achieved promising results on PD-L1 positive cases, and could significantly improve the assessment accuracy of pathologists with lower levels of relevant expertise.

**Table 1:** Baseline characteristics in the development and evaluation cohorts.

Characteristic	Development cohort	Evaluation cohort
**Age (years)**		
Average	65.1	64.6
Range	40–71	51–81
**Sex**		
Male	39	50
Female	6	1
**Stage**		
T1		18
T2		22
T3		9
T4		2
N/A	45	
**Smoking history**		
Current/former smoker		46
Never smoker		5
N/A	45	
**TPS**		
$<1\%$	2	15
1–49%	10	18
$>=50\%$	33	18

## Materials and methods

The study was approved by the institutional review boards of the participating institution, i.e., the Fudan University Shanghai Cancer Center. Informed consent was obtained from all subjects. All clinical data and digital slides used were anonymized. Methods and experiments in this study were performed in accordance with relevant guidelines and regulations.

### Materials

We collected two PD-L1 IHC WSI datasets, i.e., algorithm development cohort and evaluation cohort, for lung squamous cell carcinoma. Both were obtained from Fudan University Shanghai Cancer Center from 2018 to 2020. For each case in those two datasets, newly cut sections from formalin-fixed paraffin-embedded samples obtained by surgical resection and biopsy were used to avoid degradation of PD-L1 protein due to long-term archiving, so as to ensure the reliability of the immunohistochemical results for PD-L1 expression. It is noted that all cases in evaluation cohort were from surgical resection, while biopsies were collected for algorithm development. Two serial sections were cut from each tissue for Haematoxylin and Eosin (H&E) and PD-L1 IHC. H&E sections were stained using the Sakura Tissue-Tek Prisma staining machine (Sakura Prisma-J2S). PD-L1 IHC slides were performed on the Dako Autostainer Link 48 platform according to the automated staining protocol with the PD-L1 22C3 antibody. All slides were digitized by KFBIO FK-Pro-120 slide scanner.

The algorithm development set consists of 45 PD-L1 WSIs. According to our proposed algorithm, we extracted two different patch image datasets of TumorSeg and NucleiDetect for tumor region segmentation module and nuclei detection module respectively. TumorSeg consisted of 22,000 patch images of size $512 \times 512$ captured at $20 \times$ optical magnification ($0.475\, \upmu$m/pixel) with pixel-wise labels on tumor region. NucleiDetect contained 4600 patch images of $256 \times 256$ captured at $40 \times$ optical magnification, where nuclei centers of tumor cells were manually marked. The patch image ratios of PD-L1 expression negative to positive in both datasets were 3:7. Manual annotations were conducted by two experienced pathologists and four graduate students of pathology. Both datasets were randomly split into training and validation sets for ten-fold cross-validation.

**Figure 1:**
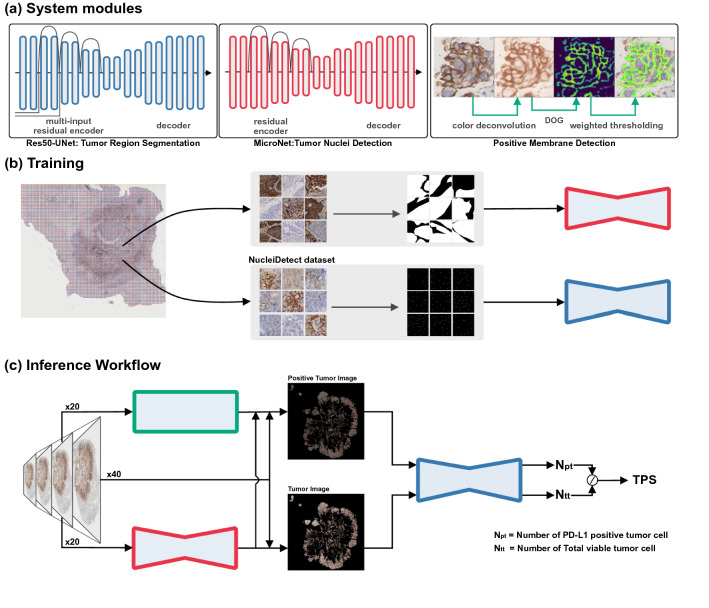
Overview of proposed framework in this study. (**a**) three main modules in ATPSS: tumor segmentation network (Res50-UNet), nuclei detection network (MicroNet), and positive membrane detection (PMD). (**b**) Model training. Res50-UNet and MicroNet were trained on datasets of TumorSeg and NucleiDetect respectively, which were cropped from WSI with different sizes and manually annotated by pathologists. (**c**) WSI inference workflow. A whole-slide image at $20\times$ resolution is fed into the trained Res50-UNet and PMD module to get the masks of tumor region and positive tumor region. $N_{pt}$ and $N_{tt}$ were predicted by MicroNet based on those two masked whole-slide images with $40\times$ resolution, and finally the tumor proportion score is produced.

A total of 51 tumor tissues from lung squamous cell carcinoma patients were collected as the evaluation cohort in this study. Inclusion criteria were that tissues contained a sufficient number of viable TCs for PD-L1 IHC testing, and the PD-L1 tumor expression of the evaluation cohort were balanced in three PD-L1 expression levels of $<1\%$, 1–49%, and $\ge 50\%$. Detailed patient demographics and PD-L1 results are summarized in Table 1.

### Automatic TPS algorithm development

We proposed an artificial intelligence based algorithm combining both deep learning and image processing techniques for PD-L1 TPS assessment. Only PD-L1 stained IHC WS image was utilized as input. The system produced two intermediate results, i.e., tumor mask and positive stain mask, and the final TPS estimation by counting tumor nuclei on those two masked regions. The overview of the automatic TPS prediction framework is shown in Fig. 1. The framework consists of three main modules: (1) *tumor area segmentation*; (2) *positive membrane detection*; (3) *nuclei detection*.

We employed the fully convolutional network (FCN) architecture named Res50-UNet to detect and segment the tumor area. The model was built on U-Net^26^, which has a symmetric encoder-decoder architecture with skip connection between downsampling and upsampling paths. We utilized the pre-trained Resnet-50^27^ as the encoder, and removed the striding in the last two blocks and applied dilated convolution with rate of 2 and 4. In addition, Atrous Spatial Pyramid Pooling (ASPP) was utilized between encoder and decoder to capture contextual information at multiple scales. The ASPP module contained four parallel atrous convolutions with increasing dilation rates of [1, 2, 4, 7].

The proposed positive membrane detection module does not require manual annotation. The DAB channel image $I_{DAB}$ was first extracted from input original RGB images using color deconvolution^28^. In order to detect the positive stained membrane, we applied a difference of Gaussian (DOG) filter on the DAB channel image, which is then processed by luminance weighted thresholding^19^. Specifically, the Luminance Adaptive Multi-Thresholding (LAMT)^29^ was first utilized to classify the positive stained pixels. We assigned luminance values to positive pixels for later thresholding. The idea was that the luminance instead of the value of $I_{DAB}$ could correctly describe the stain intensity. Finally, the positive stained region mask was obtained by post-processing with morphological operations and hole filling. The morphological operations are open and close operations, which arm to eliminate small dot noise and complete the partial membrane respectively. The positive stained cells are determined that if cells fall within the positive stain mask.

The tumor cell counting module was implemented by a nuclei detection model of Micro-Net^30^, which also resembled U-Net. The network was characterized by multi-resolution input and output for extracting multi-scale visual features. The label of the training set was processed according to repel coding scheme^31^. Finally, we used non maximum suppression (NMS) to get the final detection result for counting.

**Figure 2:**
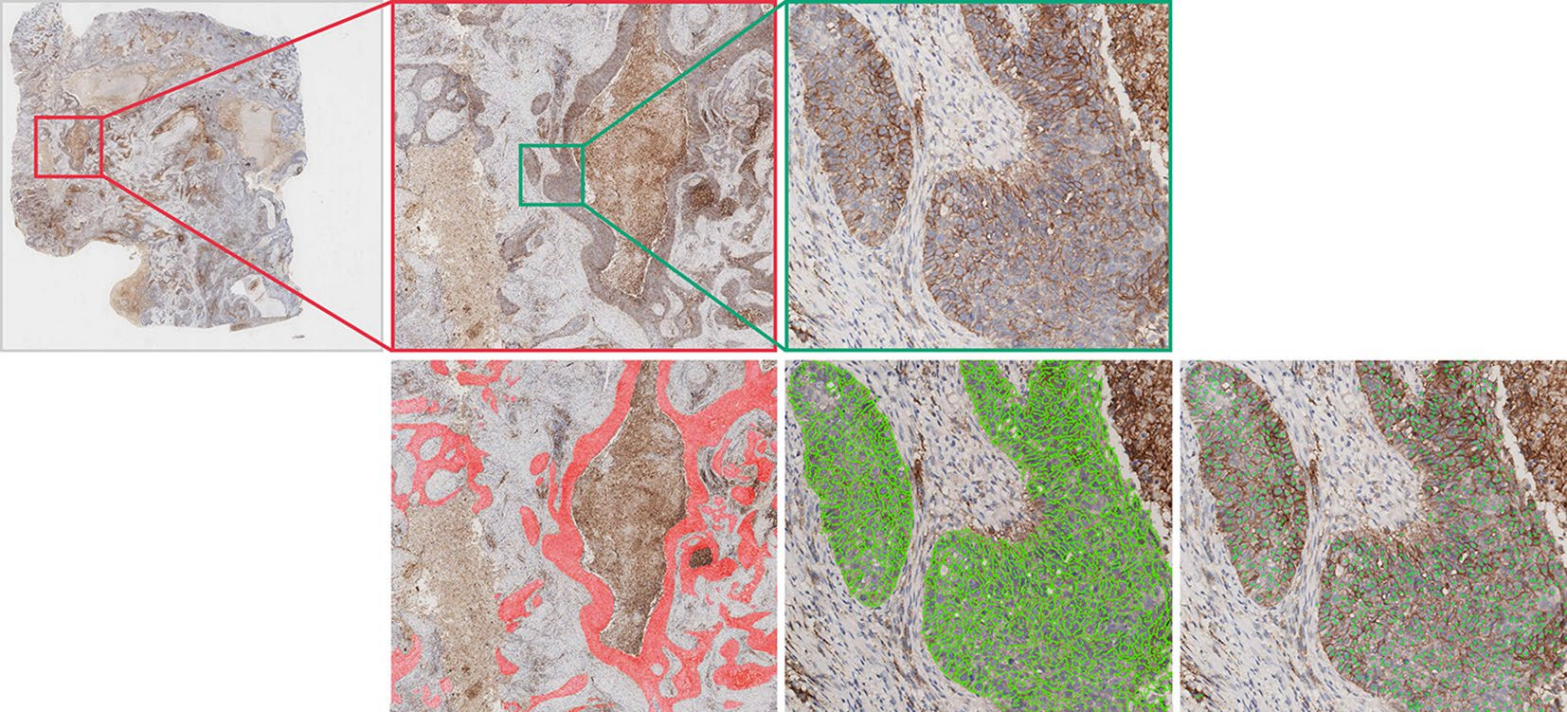
Examples of intermediate images in the ATPSS pipeline. From left to right of the top row is the original PD-L1 IHC images with different scales. From left to right of the bottom row: tumor area segmentation result, positive membrane detection result, and tumor nuclei detection result.

## Results

### Intermediate results of the proposed ATPSS

**Figure 3:**
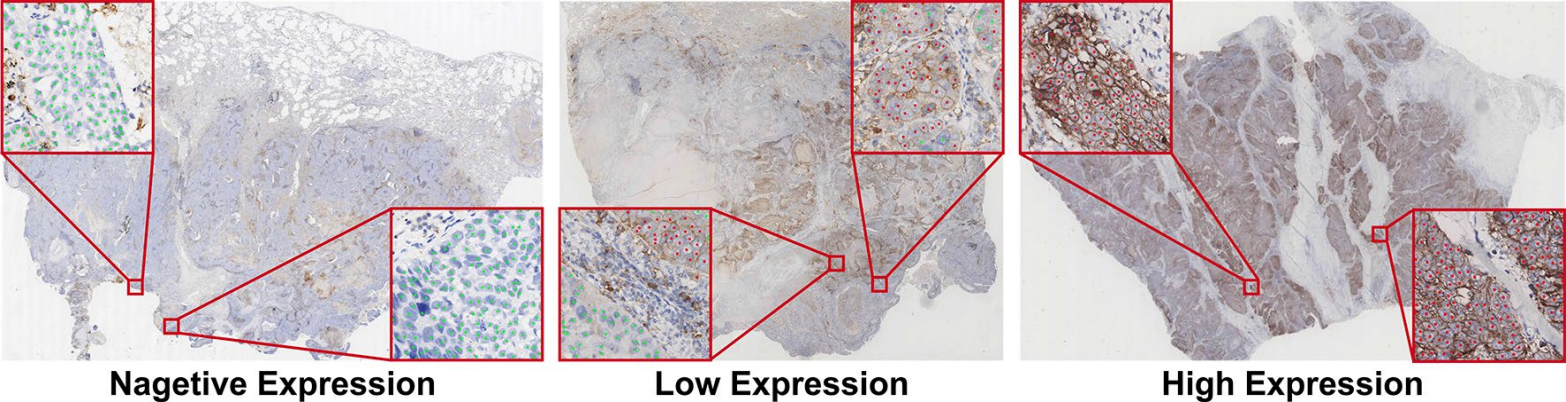
Examples of comprehensive results of proposed ATPSS that combine three intermediate results. The slides show different PD-L1 expression levels. Red dots indicate positive PD-L1 stained tumor cells, while green dots depict tumor cells negative for PD-L1 staining.

Figure 2 shows an example of the intermediate results of tumor area segmentation, positive membrane detection, and nuclei detection. To measure the performance of the tumor region segmentation model, we calculated the Overall Pixel Accuracy (OPA) and pixel level recall. The model achieved OPA of 85.66% and recall of 0.91 (95% confidence interval (CI): 79.31–90.32% and 0.8616–0.9443 respectively). The OPA in PD-L1 negative images was 82.14% while that in PD-L1 positive images was 87.02%. The segmentation result showed that the model can correctly segment the tumor regions while dismiss the necrotic and Interstitial areas on IHC images. It is seen that positive stained membranes can be clearly marked by our proposed unsupervised method. The color deconvolution combined with morphological operations was able to reliably detect and separate positive membranes. The tumor nuclei detection model was evaluated in terms of average F1 score on the validation set, where a true positive detection was determined if the Euclidean distance between the predicted point and the nearest annotated center is below 10 pixels. Using this metrics, the model achieved an area under the receiver operating characteristic (ROC) curve (AUC) of 0.901 (95%CI: 0.845–0.918) and a F1 Score of 0.859 (95%CI: 0.812–0.898). PD-L1 positive and negative tumor cell can be calculated by combining three intermediate results. The comprehensive tumor cell detection results on WSIs with different PD-L1 expression level are illustrated in Fig. 3.

### Comparison of ATPSS to pathologists on WSIs TPS assessment

In order to evaluate the impact of the automatic TPS assessment system, we designed a multi-reader multi-case study. Six pathologists involved in this study were divided into three groups based on their experience: pathology trainees (2), non-subspecialty pathologists (2), and subspecialty pathologists (2). Subspecialty and non-subspecialty pathologists have experiences of PD-L1 TPS assessment, while pathology trainees had been trained before the experiment. All pathologists can access the corresponding H&E slide during the assessment, while ATPSS only used PD-L1 IHC whole slide image for prediction. The ground truth TPS results of the evaluation cohort were from the pathology reports, which were interpreted by two subspecialty pathologists blinded to the clinical data under a light microscope (Olympus BX43).

We evaluated the results using the 3-class classification (PD-L1 expression levels of negative [<1%], low expression [1–49%], high expression [$\ge 50$%]) accuracy (ACC). Meanwhile, Mean Absolute Error (MAE) and the Pearson correlation coefficient (PCC) between the ground truth and the TPS predicted by ATPSS and pathologists were used as the evaluation metrics. As a reference, the MAE and PCC between the predicted TPS given by the two pathologists with the same experience level were also calculated.

The average accuracy for PD-L1 expression level classification of all 6 pathologists was 84.3%, and the pathologist experience level had a significant effect on TPS assessment accuracy. Among the pathologist subgroups, subspecialists was unsurprisingly accurate on all three expression levels with a classification accuracy of 97.06% and an MAE of 4.17(95% CI 3.13–5.20). The within-group correlation between two sub-specialists is 0.96 ($p<0.001$). Whereas the ACC and MAE of non-subspecialists were 84.30% and 7.91 (95% CI 5.84–9.99), and those of trainees were 71.55% and 11.22 (95% CI 8.77–13.68) respectively, which had a significant accuracy drop compared to sub-specialists (see Table 2). The within-group correlation of non-subspecialists and trainees were 0.86 ($p<0.001$) and 0.90 ($p<0.001$) respectively, and also demonstrated unreliability.

**Figure 4:**
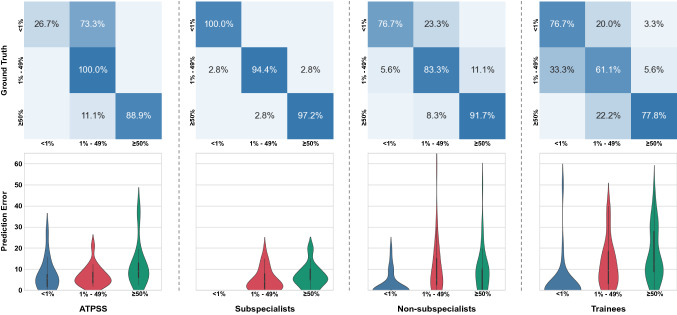
Results PD-L1 TPS assessment of ATPSS and pathologists on different PD-L1 expression levels. The top row presents the confusion matrices; bottom row shows the violin plots. The results of pathologists are the sum of two pathologists with same experience level. The ATPSS shows satisfactory level of accuracy on positive PD-L1 expression cases; the level of accuracy drops as the pathologist’s level of experience deceases.

Our further analysis found that the PD-L1 expression level also affects the TPS assessment accuracy for pathologists of all three experience levels. Subspecialists only mis-classified the PD-L1 positive WS images, and yielded MAEs of 5.08 and 6.72 for the expression levels of “1–49%” and “$\ge 50$%”. The mis-classification of non-subspecialists and trainees focus on the lower expression cases with TPS of “<1%” and “1–49%”. Non-subspecialists produced similar MAEs on two PD-L1 positive expression levels (“1–49%”: 10.61, “$\ge 50$%”: 9.72), which were worse than that of negative expression. The MAEs of trainees were increasing with the expression level of 3.05, 12.53 and 16.72. The confusion matrix and violin plot of different PD-L1 expression levels for ATPSS and each pathologist subgroup are shown in Fig. 4.

ATPSS achieved a classification accuracy of 74.51% and an MAE of 8.65 (95% CI 6.42–10.90) on the validation cohort, which was better than trainees and worse than non-subspecialists. It can be found that ATPSS gave poor prediction on negative PD-L1 expression cases, which mistakenly classified the negative expression into low expression level (Fig. 4). We excluded the negative PD-L1 expression cases, the MAE and classification accuracy of ATPSS were 9.3%(6.57–12.07) and 94.4%, respectively, which significantly surpassed those of non-subspecialists (MAE: 10.17 [95% CI 7.49–12.85], ACC: 87.5%) and trainees (MAE: 14.63 [95% CI 11.82–17.44], ACC: 69.4%). Within PD-L1 expression subgroups, the MAE of ATPSS were 7.06, 6.73 and 11.90 for “<1%”, “1–49%”, and “$\ge 50$%” respectively. While ATPSS produced significantly lower MAE than that of the non-subspecialist group in “1–49%”, and their performance is similar in “$\ge 50$%”.

**Table 2:** The performance of ATPSS and pathologists for PD-L1 tumor proportion score assessment.

	ACC (%)	MAE [TPS%]				PCC (*p* value)
		Overall (95% CI)	<1%	1–49%	>50%	
ATPSS	74.51	8.65 (6.42–10.90)	7.06	6.73	11.90	0.9436 ($<0.001$)
Subspecialist	97.06	4.17 (3.13–5.20)	0.00	5.08	6.72	0.9828 ($<0.001$)
Non-subspecialist	84.30	7.91 (5.84–9.99)	2.50	10.61	9.72	0.9268 ($<0.001$)
Trainee	71.55	11.22 (8.77–13.68)	3.05	12.53	16.72	0.8934 ($<0.001$)

## Discussions

In this study, we developed an automatic TPS assessment algorithm named ATPSS for IHC PD-L1 whole-slide images of squamous cell lung cancer patients, which is highly helpful to improve pathologists’ diagnosis accuracy and efficiency. The ATPSS utilized widely proved deep learning models and image processing techniques, and the intermediate results were calculated according to pathologists’ assessing process.

According to the results of ATPSS and the histopathological manifestations of the cases, we can conclude that ATPSS was able to accurately distinguish tumor parenchyma, interstitium, most infiltrating immune cells and tissue cells in lung squamous cell carcinoma, and could precisely identify PD-L1 membrane-positive tumor cells from histiocytes, which showed a high level of agreement with the ground truth. In addition, the performance of tumor region segmentation was consistent on the IHC images with different PD-L1 expression levels. With the uniform thresholds of DAB stains, the positive membrane detection module showed more precise results than pathologists.

**Figure 5:**
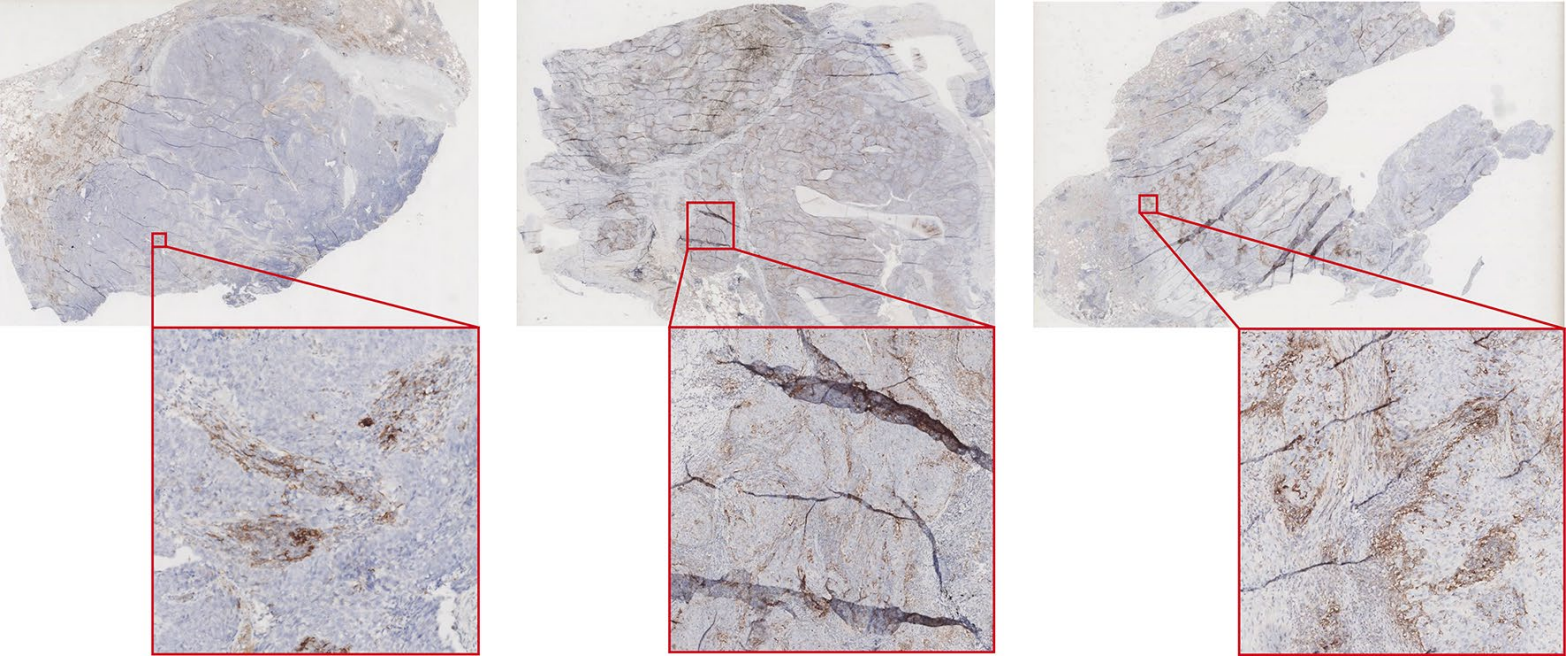
Three PD-L1 negative expression slides mis-classified as weak expression. These slides have different levels of tissue-fold, non-specific staining, and intermixing of tumor and non-tumor region.

However, ATPSS easily mis-classified the PD-L1 negative cases with TPS <1% as low PD-L1 expression of TPS 1–49% (see Fig. 5). Among the 11 cases that were mis-classified as level of 1–49%, most of the cases (8/11, 72.7%) were interpreted to have a TPS ranging from 1 to 10%. Those PD-L1 IHC images were individually analyzed by thoracic subspecialty pathologist, and the main reasons can be summarized: (1) a small number of membrane-positive histiocytes or other infiltrated immune cells scattered inside and around the tumor nests were recognized as positive tumor cells; (2) a few of necrosis or apoptotic foci in tumor nests were recognized as positive tumor cells due to the non-specific staining.

The last three negative PD-L1 expression cases were assessed with TPS ranging from 15.78 to 27.8% by ATPSS. There were two reasons leading to the large deviation. Firstly, poor tissue processing and staining quality, such as poor immunohistochemical staining, peeling off, tissue-fold, etc, lead to the poor elution of second antibodies and chromogenic reporter, resulting in non-specific staining in many areas, which directly affected the performance of tumor region detection and tumor cell detection. Secondly, those cases had common characteristics, i.e., PD-L1 positive non-tumor cells are closely intermixed with tumor nests, or distributed in clusters on the edge and inside of tumor nests. Furthermore, those cases with large deviation usually showed complex histologic architecture due to poor differentiation, such as poorly cohesive pattern and single cell stromal invasion. Such complex architecture and mixed immune infiltration pattern results in identification mistakes, e.g. mis-recognizing positive non-tumor cells as positive tumor cells.

It should be noted that similar problems of the non-specific staining and complex cancer tissue structure also occurred in the positive expression slides. Nevertheless, such incorrectly identified area was significantly smaller than the whole tumor area, which would not have a great influence on the final TPS assessment. The main causes of large assessment error by ATPSS on positive PD-L1 expression cases were low image quality, such as out-of-focus, tissue-fold, and unclear nuclei caused by strong DAB staining. Therefore, it is of great significance to control the quality of samples submitted for inspection in future works.

The study has several limitations. From the perspective of model development, the unbalanced development cohort was also a possible reason of poor accuracy on PD-L1 negative cases. Although two datasets (e.g., TumorSeg and NucleiDetect) had a sufficient amount of PD-L1 negative patch images from PD-L1 positive cases, the morphological particularity of the PD-L1 positive cases were ignored. Therefore, a balanced development cohort may be one of the possible solutions to this problem. The ground truth of the evaluation cohort was assessed manually by pathologists without accurate quantification of tumor areas or tumor cells. As a result, the TPS values have a quantitative step-size of 5. If a WS image was assessed at 70%, it means that the image has a value of around 70%. However, as a fully automatic and quantitative assessment system, the ATPSS predicted TPS at cell level. Therefore, in the strict sense, the ground truth cannot be used as the gold standard for validating ATPSS performance. More accurate ground truth can be obtained by summarizing several experienced pathologists’ results or manually correction of ATPSS’s intermediate results with computer assisted system. According to the suggested methods for determining TPS provided by ‘*PD-L1 IHC 22C3 pharmDx Interpretation Manual - NSCLC*’^13^, the TPS assessment on the whole slide images are based on selected tumor areas. Therefore, instead of whole slide image assessment, the tile-based assistant experiment (e.g. pathologists first visually divide the tumor area into tiles with equal size, and select several tiles for TPS prediction) may be closer to the clinical diagnosis process. Finally, our study was limited to the cases from a single hospital of Fudan University Shanghai Cancer Center. A further study incorporating larger datasets from multiple hospitals, digitized with different scanners, as well as more pathologists with border experience levels will be necessary to validate the automatic tumor proportion scoring system.

Future work can further develop this research in aspects: biopsy ability, adenocarcinoma support, and computer assisted system. First, routine diagnostics of NSCLC are typically needle biopsy. To develop the ability on biopsy, a specialized dataset should be collected for training the tumor area segmentation model, and the model should also have a larger receptive field for extracting long range morphological information. Second, in this work, we only study the squamous cell carcinoma of NSCLC. Adenocarcinoma has a more complex morphology of tumor compared to squamous cell carcinomas. Our preliminary study found that manual annotation is time consuming and has serious inter-difference between different pathologists. To solve this, future research can employ manual correction based on the transfer result of the tumor area segmentation model of ATPSS to accelerate the annotation process. Finally, future research can further develop computer assisted system which combines the results of artificial intelligence based algorithm and pathologists for more accurate TPS assessment.

In summary, we have developed computer-aided system using IHC PD-L1 whole slide images of lung squamous cell carcinoma for automatic tumor proportion scoring. ATPSS was able to achieve high assessment accuracy on positive PD-L1 expression cases and significantly surpass to that of non-subspecialists and trainees. In doing so, we show the potential of automatic or computer assisted tumor proportion scoring to improve the effectiveness and accuracy of quantitative PD-L1 assessment.
